# Celiac Disease, Gluten-Free Diet, and Metabolic and Liver Disorders

**DOI:** 10.3390/nu12040940

**Published:** 2020-03-28

**Authors:** Marco Valvano, Salvatore Longo, Gianpiero Stefanelli, Giuseppe Frieri, Angelo Viscido, Giovanni Latella

**Affiliations:** Gastroenterology, Hepatology and Nutrition division, Department of Life, Health and Environmental Sciences, University of L’Aquila, 67100 L’Aquila, Italy; valvano.marco@libero.it (M.V.); salvator.longo@gmail.com (S.L.); giastefanelli@gmail.com (G.S.); g.frieri@libero.it (G.F.); angelo.viscido@univaq.it (A.V.)

**Keywords:** celiac disease, gluten-free diet, hepatic steatosis, nonalcoholic steatohepatitis, NASH, nonalcoholic fatty liver disease, NAFLD, dyslipidemia, hypercholesterolemia, hypertriglyceridemia, body weight, body mass index, BMI, type 2 diabetes mellitus, T2DM

## Abstract

Celiac disease (CD) is a chronic autoimmune enteropathy triggered by the ingestion of gluten in genetically predisposed individuals. At the time of diagnosis, the frequency of nonalcoholic fatty liver disease (NAFLD) and nonalcoholic steatohepatitis in individuals with CD appears to be similar to that of the general population, although a lower body mass index and a lower rate of hypercholesterolemia and type 2 diabetes mellitus are observed at diagnosis in CD patients. The effect of a gluten-free diet (GFD) in individuals with these liver and metabolic disorders is still a matter of debate. The aim of this study was to investigate the links between a GFD and metabolic/liver disorders in CD patients. A systematic electronic search of the literature from January 2009 to December 2019 was performed using Medline, Web of Science, Scopus, and the Cochrane Library. Only papers written in English concerning metabolic and liver disorders in adult patients with CD were included. Out of 1195 citations, 14 eligible studies were identified. Increases in the frequency of NAFLD, weight gain, and alterations of the lipid profile suggest that important changes happen in celiac patients on a GFD, though the physiopathology of these conditions is unclear. Although a GFD is the only effective treatment available for CD, liver function, body weight, and metabolic and nutritional profiles should be monitored in patients on a GFD.

## 1. Introduction

Celiac disease (CD) is a chronic autoimmune enteropathy triggered by the ingestion of gluten in genetically predisposed individuals: it induces damage to the mucosa of the small intestine, which is characterized by atrophy of the villi [1]. The clinical manifestations are heterogeneous and include both "classical CD" (symptoms of malabsorption, e.g., diarrhea, steatorrhea, and weight loss) and "nonclassical CD" (extraintestinal symptoms, e.g., anemia, osteoporosis, liver disorders, and depression) [1,2]. 

Historically, at the time of diagnosis, patents with CD had a reduced body mass index (BMI) compared to the general population [3]. In the last few decades, increasing numbers of CD patients with a normal or high BMI at the time of diagnosis was reported [4,5,6,7,8,9]. This could be due to the fact that early diagnosis has become more frequent in recent years (late diagnoses were more common in the past), allowing for a reduction in the rate of both intestinal mucosal atrophy and related malabsorption [9]. Furthermore, the milder forms of CD (those which do not get worse by time) are increasingly recognized.

The mean BMI in CD patients at diagnosis is lower than in the general population (22.4–24.8 vs. 24.2–27.9, respectively) [10,11,12]. However, although the frequency of CD patients being overweight is lower than in the general population, overweight patients are increasingly being found at diagnosis. It has been observed that the nutritional status of patients who have received a diagnosis of CD in the last few years is better than that of those diagnosed in previous years. In one study, out of 155 patients diagnosed with CD between 1994 and 2017, patients who were overweight at diagnosis were observed only after 2002, and obesity was observed only after 2010 [9]. 

The clinical presentation of CD has changed over time, with "nonclassical CD" (extraintestinal symptoms) being more common than classical symptoms of malabsorption [13]. Out of the atypical manifestations of CD, liver involvement is among the most common [14]. In a meta-analysis including eleven studies, pooled prevalences of positive celiac serology and biopsy-proven CD in cryptogenic hypertransaminasemia were 6% (95 CI 3% to 10%) and 4% (95% CI 1% to 7%), respectively [15]. Pooled prevalence of hypertransaminasemia in newly diagnosed CD was 27% (95% CI 13% to 44%). Moreover, different prevalences of elevated levels of transaminases were reported. In a cross sectional study including 313 untreated and 339 treated adult patients with CD and 237 non-celiac controls, the proportion of patients with hypertransaminasemia in the untreated CD group (11%) did not differ significantly from that in the treated CD (8%) or non-celiac control group (9%) (*p* < 0.587) [16]. More recently, it has been reported that approximately 40% of patients had elevated liver function tests (LFTs) at CD diagnosis and that the majority were then nominalized after a gluten-free diet (GFD) [10]. LFTs were defined as abnormal liver enzyme levels based on the Third National Health and Nutrition Examination Survey III criteria (NHANES III). Gluten-sensitive hypertransaminasemia in celiac disease should be considered as an infrequent and often subclinical finding. The values of transaminases usually normalize within 6 to 12 months after the patient starts a gluten-free diet (GFD) [17]. 

On the other hand, the frequency of nonalcoholic fatty liver disease (NAFLD) and nonalcoholic steatohepatitis (NASH) in CD patients at the time of diagnosis appears to be similar to that of the general population (5.4% vs. 4.5%, *p* = 0.545) [10]. 

NAFLD is a common condition in the general population, and its prevalence is extremely heterogeneous all over the world. In Europe, its frequency ranges from 4% to 49.62% (overall prevalence of 23.71%; confidence interval 16.12%–33.45%). The prevalence of NASH is lower, ranging from 3% to 5% [18,19]. Furthermore, NAFLD patients have a higher risk of a new diagnosis of CD compared to the general population [20,21], despite celiac patients having a lower risk for metabolic syndrome before the start of a GFD [20]. 

In fact, the prevalence of type 2 diabetes mellitus (T2DM) at diagnosis in celiac patients is lower or similar to that of the general population (2.7%–3.1% vs. 3%–9.6%) [22,23]. In a UK study, CD patients showed a low rate of hypercholesterolemia compared to the general population (3.0% vs. 4.8%; odds ratio-OR: 0.58) [24].

The effect of a GFD in individuals with these conditions is still a matter of debate. It has been hypothesized that gluten-free foods could play a key role in the etiology of metabolic and hepatic disorders. It has been demonstrated that the consumption of gluten-free products leads to higher lipid and carbohydrate intake [25,26,27,28]. However, it is not clear if gluten-free products contribute to weight gain, and furthermore, the role of a GFD in the development of NAFLD remains undetermined.

The aim of our study was to investigate the links between a GFD and metabolic/liver disorders in CD patients. In particular, we investigated the association between changes in body weight and the lipid profile and the frequency of T2DM and NAFLD before and after the start of a GFD.

## 2. Materials and Methods 

A systematic electronic search of the literature (written in English) from January 2009 to December 2019 was performed using Medline (PubMed), Web of Science, Scopus, and the Cochrane Library. The search included a combination of Medical Subject headings (MeSH) and keywords: 

“celiac disease/coeliac disease”, “steatosis”, “hepatic steatosis”, “steatohepatitis”, “nonalcoholic steatohepatitis”, “NASH”, “nonalcoholic fatty liver disease”, “NAFLD”, “dyslipidemia”, “hypercholesterolemia”, “hypertriglyceridemia”, “weight gain”, “body mass index”, “BMI”, and “type 2 diabetes mellitus”.

The search was limited to studies conducted on human, adult CD patients on a GFD. Studies on pediatric populations (0–16 years) were excluded. We considered prospective and retrospective cohort studies, case–control studies, and analytical cross-sectional studies. 

## 3. Results

**Figure 1:**
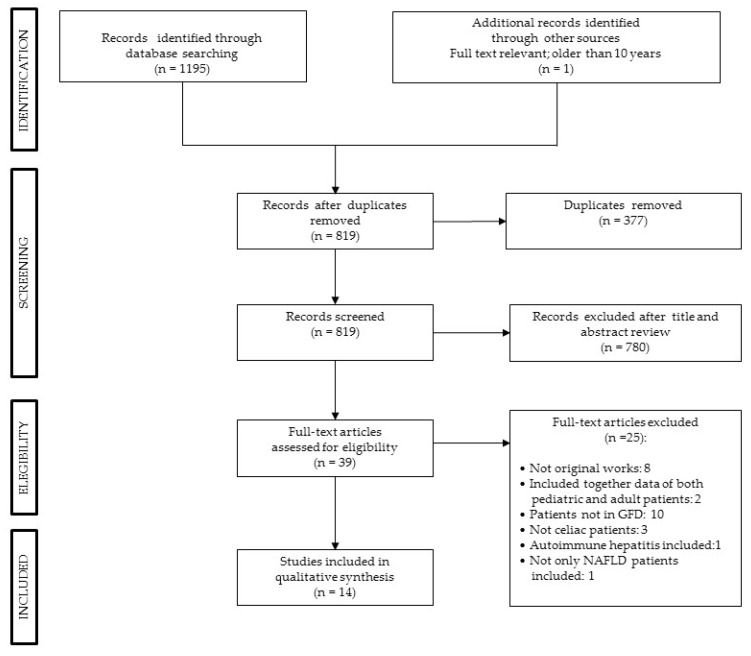
PRISMA flow diagram.

Figure 1 shows the results of the literature search, as assessed by the three authors (M.V., S.L., and G.S.). We found 1195 articles, removing 377 duplicated records, excluding 780 records based on their titles and abstracts. Two studies were excluded because they included both pediatric and adult patients and data of the two groups were not reported separately, therefore data from adult CD patients could not be extrapolated. For this review, we selected and included 14 articles that reported only data of adult patients with CD on a GFD. The characteristics of the 14 selected studies are reported in Table A1 in Appendix A.

### 3.1. Celiac Disease and BMI While on A GFD

Nine studies assessed the BMI changes in CD patients before and after starting a GFD [4,11,29,30,31,32,33,34,35]. Six of these studies analyzed BMI changes as expressed by the mean or median (Table 1) [11,29,30,31,32,33], while the remaining three studies evaluated the number of patients with changes in their BMIs in terms of a percentage [4,34,35]. 

A prospective study including 39 CD patients and 39 controls, with two years of follow-up, showed that patients with a normal BMI (18.5–24.9 Kg/m^2^) at diagnosis gained weight compared to the control group [29]. However, despite the weight gain, patients with a normal BMI did not move to the overweight or obese groups (BMI ≥ 25 Kg/m^2^), while on the other hand, 50% of underweight (BMI < 18.5 Kg/m^2^) patients switched to the normal weight group. We note that a potential bias of this study was that these patients were on a Mediterranean-type diet, which is different from a Western diet. The latter is high-calorie diet rich in fats and sugars and can alter gut microbiome composition and metabolic activity [29].

A retrospective study involving 369 CD patients showed that a GFD had a beneficial effect on BMI during the follow-up period [11]. The Authors observed that after starting a GFD, about 60% of patients with a BMI < 18.5 Kg/m^2^ gained weight, and 41% of overweight and obese patients lost weight. However, the majority of patients had a normal BMI at diagnosis, and about 47% gained weight. They also observed that presentation with diarrhea, being female, and more severe villous atrophy (Marsh IIIb/c) were predictor factors for a low BMI at diagnosis [11].

In a retrospective study of 679 celiac patients on a GFD with a longer follow-up period (mean of 39.5 months; range 1–345), 15.8% moved from a normal or low BMI class into an overweight class (BMI > 25 kg/m^2^) after starting a GFD, and 22% of overweight patients gained weight after starting a GFD [30]. Overall, the mean BMI increased significantly with a GFD, and in the group of patients with a normal BMI at the beginning of the study, 21.8% of patients gained weight and increased their BMI by more than two points. A BMI decrease of at least two points was observed in 4.8% of patients (*p* = 0.0001) [30].

Moreover, an overall increase in BMI after starting a GFD was observed in three other studies (Table 1) [31,32,33]. 

In a prospective cohort of 698 patients with a recent diagnosis of CD, the changes of BMI were evaluated [34]. In this study, 677 out of 698 patients on a GFD were included in a one-year follow-up: 33% gained at least 3 kg and 16% lost at least the same amount of weight. Notably, 22% of those who were overweight and 38% of those with a normal weight gained weight during the follow-up period (> 3 kg). It is important to note that this was a prospective study and therefore it could have induced patients to adopt a more balanced diet and a healthier lifestyle. Finally, weight and height were self-reported, which might have led to an underestimation of BMI [34]. 

In a retrospective study with a longer follow-up period (2 years) that included patients on a GFD with a new diagnosis of CD, 81% gained weight, including 82% of initially overweight patients [4]. In another retrospective study, the number of patients with a BMI > 25 kg/m^2^ increased significantly after the initiation of a GFD (45/185 vs. 78/185; *p* = 0.0001; follow-up of 7 years; range 1–36 years) [35].

### 3.2. Dyslipidemia in Celiac Disease Following A GFD 

There were three studies found that assessed lipid levels before and after the initiation of a GFD (Table 2) [32,33,35] and one study that reported the prevalence of dyslipidemia in celiac patients on a GFD [22]. In a case-control study, increasing triglyceride blood levels were observed after the diagnosis of CD in patients following a GFD at a follow-up of 4.7 years [33]. In another prospective study, however, the same authors did not find significant changes in the levels of triglycerides (although this was only after a one-year follow-up) [32]. In a retrospective cohort of 185 patients with CD, an increase in the prevalence of hypercholesterolemia and a reduction in the high-density lipoprotein (HDL) level was observed; however, the increase in hypertriglyceridemia was not statistically significant (*p* = 0.339) [35]. The Authors reported a worsening of the lipidic profile during the follow-up, which included a higher total cholesterol level, more triglycerides, and a decrease in the HDL level (Table 2) [35]. On the other hand, in a cross-sectional study, the prevalence of dyslipidemia in patients with CD was significantly lower compared to the control group (18.3% vs. 34.9%; *p* < 0.0001) [22].

### 3.3. Celiac Disease Following A GFD and Type 2 Diabetes Mellitus

There were two studies found that assessed the prevalence of T2DM in CD patients on a GFD (Table 3) [22,36], and two studies examined the glycemia level before and after the start of a GFD [32,33].

In a cross-sectional study involving 840 CD patients and 840 healthy subjects, the prevalence of T2DM was three times lower in the celiac group than in the matched control and in the general population [22]. Furthermore, CD patients had a lower BMI (24.7 vs. 27.5 Kg/m^2^; *p* < 0.0001). The incidence of T2DM seemed to not be influenced by the duration of the GFD. In fact, in patients that were on a GFD for less than two years, the prevalence of T2DM was not statistically different from the prevalence in those on a GFD for more than two years, despite the fact that the patients on the GFD for longer were older (1.4% vs. 3.3%; *p* = .7) (Table 3). Whether a lower BMI in celiac patients is a protective factor for the incidence of T2DM is not clear, the mechanisms by which patients with CD had lower prevalence of T2DM are not clear, however this finding could be attributable to altered pancreatic function, impaired nutrient absorption, and changes in gastrointestinal endocrine functioning. Furthermore, tissue transglutaminase drives inflammation in CD, impairing the expression of peroxisome proliferator-activated receptor *γ*, which could be implicated in a decreased risk of T2DM [22].

In a large population-based cohort of 26,816 patients with CD and 130,051 controls the prevalence of T2DM found in celiac patients on a GFD was the same of that observed in matched reference individuals (Table 3) [36]. A critical point of this study is the control group of patients including a very different population. 

With regard to glycemia levels, a prospective study showed significant differences at CD diagnosis and follow up [32]. Of the included 98 patients with CD, 7 patients (7.1%) at CD diagnosis versus 25 patients (25.5%) after starting GFD had blood glucose levels which exceeded the threshold (*p* < 0.01, OR 3.7). The mean glycemic value after 1 year of GFD was 92 mg/dL versus 86 mg/dL at CD diagnosis [32]. In another case-control study, the same Authors reported an increase in the fasting blood glucose levels after the start of a GFD compared with those at CD diagnosis (mean ± SD: 88.7 ± 13.4 mg/dL vs. 84.1 ± 19.8 mg/dL, respectively) [33]. 

### 3.4. Celiac Disease Following a GFD and NAFLD

There were four studies found that assessed the frequency of NAFLD/NASH before and after the start of a GFD (Table 4) [35,36,37,38]. Three of them evaluated any predictive factors that could increase metabolic risk during the GFD [19,33,38]. 

In a prospective study, patients with celiac disease had a major risk of developing NAFLD compared to the general population [36]. The risk was highest in the first year after diagnosis and was persistently higher than in the general population over the next 15 years. This work involved a cohort of 26,861 patients with CD with no signs of liver disease at diagnosis (and no history of alcohol-related disorders). This cohort was compared to a group of 130,051 healthy control patients from the Swedish national registry. The risk of developing NAFLD in celiac patients was significantly elevated compared to the general population. The hazard ratio (HR) was higher in the first year after celiac disease diagnosis, with a 4.2-fold increase in the first 5 years, and was significantly higher in the male population (male 4.5, 95% CI = 2.7–7.6 vs. female 2.1, 95% CI = 1.3–3.2) (Table 4) [36]. 

Similar results were reported in a case–control study involving 202 patients with CD on a GFD for at least six months [37]. Patients with CD were compared to control patients matched based on demographic characteristics (age and gender) and metabolic risk factors (BMI, Diabetes Mellitus, triglycerides, and cholesterol). The prevalence of NAFLD was significantly higher in the celiac population (34.7%) compared to the controls (21.8%) (*p* = 0.006). Binary logistic regression confirmed an increased risk of NAFLD in patients with CD (adjusted OR = 2.904; CI 1639–5146; *p* < 0.001). The authors also observed that the raw prevalence of NAFLD in CD patients with normal weight was even higher than in the general population (20% vs. 5.8%, respectively; *p* < 0.001). The multiple logistic regression also showed a higher relative risk for NAFLD in these celiac subjects with a normal weight (adjusted OR 5.713; CI 2300–14194; *p* < 0.001). On the other hand, in the overweight population (BMI > 25 kg/m^2^), the prevalence of NAFLD in the CD patients and controls was not significantly different (67.8% vs. 55.4%; *p* = 0.202). The Authors suggested that traditional risk factors could influence the occurrence of NAFLD in celiac subjects, as they do in the general population. On the other hand, the celiac patients developed NAFLD even when they had fewer risk factors than the general population. Therefore, the effect of a GFD on the pathogenesis of liver damage could have been underestimated [37].

In a retrospective cohort of 185 patients with CD, the incidence of hepatic steatosis (HS) in celiac patients at diagnosis and during follow-up was assessed [35]. HS was found by ultrasound in three patients (1.6%) at CD diagnosis. At the end of the follow-up period (median = 7 years; range 1–36), the prevalence of HS (*n* = 20; 11.0%) was significantly higher than at the time of CD diagnosis (1.6%) (*p* < 0.001) [35]. 

A prospective study involving 301 patients on a GFD showed the same results. The Authors observed that 112 out of 301 (37.2%) patients developed HS over a one-year follow-up period [38] (Table 4). 

In a large prospective longitudinal study, the frequency of CD among NAFLD patients and clinicopathological pattern and outcome was assessed [19]. CD was found in 7.2% of patients with NAFLD. The concomitant presence of CD and NAFLD showed a high degree of intestinal damage at diagnosis. Patients with concomitant CD and NAFLD achieved similar clinical improvement after adherence to GFD. However, intestinal histological improvement was less and delayed in patients with concomitant CD and NAFLD. Moreover, at the end of the follow-up period, more patients with concomitant NAFLD and CD progressed to NASH than patients with NAFLD alone. Therefore, the authors suggested that the two diseases had a negative impact on each other [19]. 

Finally, in a prospective cohort of 301 patients with CD, predictive factors for metabolic syndrome (MS) and HS in patient with CD after 1 year of GFD were evaluated [38]. Of the 301 CD patients, 4.3% met the criteria for MS diagnosis and 25.9% showed HS at the time of CD diagnosis. After 1 year of GFD, 23.9% developed MS (4.3% versus 23.9%; *p* < 0.001; OR 6.9) and 37.2 developed HS (25.9% vs. 37.2%; *p* < 0.01; OR 1.69). At multivariate analysis, high BMI at CD diagnosis (OR 10.8; *p* < 0.001) and use of proton pump inhibitors (PPIs) (OR 22.9; *p* < 0.001) were the only factors associated with the occurrence of MS. Insulin resistance (OR 9.7; *p* < 0.001) and PPIs exposure (OR 9.2; *p* < 0.001) were the only factors associated with the development of HS [38]. 

## 4. Discussion

The studies included in this review showed an increased prevalence and incidence of NAFLD and weight gain, a worse lipid profile, and high blood glucose levels, suggesting that important changes occur in celiac patients on a GFD [4,11,19,22,29,30,31,32,33,34,35,36,37,38].

The mechanisms leading both CD and GFD to the metabolic alterations such as the increase in body weight and BMI, blood triglyceride and cholesterol levels and blood glucose levels, as well as the development of NAFLD, remain to be clarified. These metabolic alterations could be due either to the increase in nutrient absorption processes after the start of GFD or to the consumption of a high-calorie diet rich in fats and simple carbohydrates (sugars). The regression of inflammation and atrophy of the gut mucosa induced by GFD can led to a marked improvement of intestinal absorption of nutrients in subjects who are in a compensatory hyperphagic status [32]. This is indirectly confirmed by the fact that in patients with CD a more severe degree of villous atrophy and the concomitant diarrhea were independent predictors for a lower BMI [11]. Non-adherence to the GFD appears to contribute to weight loss, while adherence may be an important factor in determining weight gain in CD patients. In addition, the duration on GFD was found to be a possible variable affecting changes in BMI [30]. 

Several studies demonstrated that GFD, although it is the only available treatment for CD, has potentially negative effects on nutritional and metabolic status [33,39]. A British study reported that female CD patients consumed significantly more energy from all macronutrients when compared with a nonceliac population, attributing this to higher intake of sweet snacks that were richer in saturated fat [40]. CD patients also had significantly lower intakes of fibers.

There is evidence that GFD can determine a higher intake of simple sugars, proteins and saturated fat and a lower intake of complex carbohydrates and fibers [32,41]. In patients with CD on a GFD, a higher intake of lipids as compared with controls was found, confirming that long-term GFD may not be nutritionally balanced [29,42,43]. In addition, many gluten-free foods are characterized by a higher glycemic index than that of equivalent gluten-containing foods [27,44]. It is probable that the absence of gluten on the one hand increases the amylase’s ability to digest starch in the gut lumen, therefore increasing starch absorption, and on the other determines the unpalatability of some gluten-free foods, then inducing a preference towards hyperproteic and hyperlipidemic foods [29,41,45].

In patients with CD on a GFD, both the increase in nutrient absorption (as a result of the improvement of the atrophy of the gut mucosa) and the intake of higher calories, fats and simple carbohydrates could contribute to the worsening of the metabolic status and to the increase of the prevalence of NAFLD in these patients [36,37]. 

Recent evidence has suggested that, despite a lifelong gluten-free diet, some disorders can persist, e.g., increased gut permeability, small bowel overgrowth, microbiota changes, exocrine pancreatic insufficiency, and low-grade gastrointestinal inflammation [46,47,48,49]. Some of these disorders could be responsible for the development of NAFLD. Of these factors, the most studied was the mucosal barrier, in particular the intestinal permeability. 

Several studies have shown that intestinal permeability may play a role in the pathogenesis of NAFLD [50]. In fact, some endotoxins, such as lipopolysaccharides (common endotoxins of gut bacteria), could reach the liver and trigger an immune reaction through the “Tall-like receptors” [51]. The reduction in the incidence of NAFLD/NASH after the start of a GFD could be related to the improvement of gut mucosal inflammation, atrophy, and permeability induced by the diet. Whether a compromised intestinal barrier is a cause or effect of NAFLD requires additional study, though the prevailing theory is that bacteria derived endotoxin and related cytokines may play a central role in the evolution of liver steatosis in NASH. Furthermore, in addition to the previously described mechanisms, a gluten-free diet itself could have a role. In the last few years, the nutritional profile of gluten-free food products has been increasingly questioned within the scientific community. The nutritional quality of the gluten-free food products currently available on the market is inadequate –low protein contents and high fat, sugar and salt contents–compared to equivalent gluten-containing products [52,53,54]. Data from studies on gluten-free foods have indicated that there is a need to lower fat and calories and to include more fibers, proteins, electrolytes, vitamins, and other micronutrients. Another important factor that is usually not taken into consideration is the market for gluten-free foods and the economic strength of CD patients. Gluten-free food availability has increased in most markets, especially in more expensive ones. The lower availability and quality of the gluten-free foods sold in cheaper markets could disproportionately impact poor socioeconomic cohorts [55]. Finally, a hyperphagic compensatory status usually follows malabsorption disorders and induces weight gain [37].

All of the above-mentioned factors could contribute to the development of NAFLD in CD patients on a gluten-free diet. It is important to underline that NAFLD is considered to be a potential hepatic expression of MS [35]. MS involves interrelated risk factors for cardiovascular disease and diabetes. These factors include hypertension, low HDL cholesterol levels, elevated triglyceride levels, diabetes/insulin resistance, and central adiposity. The pathophysiology of these conditions is unclear, but several hypotheses have been proposed. 

Most studies in the literature have shown that CD patients (at diagnosis) have a lower BMI than the general population [10,11,12]; therefore, weight gain from a GFD may have positive effects on these patients. However, it has been clearly demonstrated that nowadays, an increasing number of CD patients present with a normal or high BMI at the time of diagnosis [3,4,5,6,7,8,9]. 

It is important to underline that, despite improvements in body weight (weight gain in underweight patients and weight loss in overweight patients), patients with a normal weight usually gain weight. In fact, an increase in the average BMI was observed in all studies found (Table 1) [19,20,21,35,36,37]. In patients with CD on a GFD, the increase in BMI is probably linked to the increased intake of calories, fats and simple carbohydrates, but probably also to reduced physical activity. More than half of women with celiac disease frequently report low physical activity both before and after a GFD [56]. The reduced physical activity could be linked to the chronic fatigue which is a common finding in patients with CD, which ameliorates with the GFD [57]. Increased body weight and BMI can represent a risk factor for both NAFLD and T2DM.

Therefore, the global epidemic of obesity and NAFLD, increased BMI values at CD diagnosis, and the impact of a GFD on the metabolic status of these patients could be important and could change historical concepts of the malnutrition status of celiac patients.

T2DM per se does not appear to be increased in patients with CD compared with controls. Only in two studies, a higher prevalence of hyperglycaemia was reported in patients with CD after GFD compared to that observed at CD diagnosis. In none of these studies the current criteria for the diagnosis of T2DM had been specified and adopted; diabetes may be diagnosed based on plasma glucose criteria, either the fasting plasma glucose (FPG) value or the 2-h plasma glucose (2-h PG) value during a 75-g oral glucose tolerance test (OGTT), or HbA1C criteria [58]. Therefore, GFD would appear to induce only a slight increase in fasting blood glucose levels, but not a real increase in the prevalence of T2DM. In some cases the diagnosis of T2DM was made before the CD diagnosis, therefore GFD had no role in the development of diabetes [22].

Although NAFLD and T2DM are often found together in adults, in patients with CD the increase in prevalence in NAFLD not associated with diabetes can be detected [35,36,37,38]; at most NAFLD seems to be associated only with a slight increase in fasting blood glucose levels [32,33].

The incidence of T2DM is known to increase with age and BMI, and is linked closely to both diet and ethnicity. The increase in body weight and BMI seen in patients with CD after GFD could theoretically be a risk factor for both NAFLD and for T2DM, but the latter does not seem to be increased in the CD, either at diagnosis or after GFD [22,36].

NAFLD has been predominantly linked to obesity, dyslipidemia, and insulin resistance, although NAFLD can occur in those with a normal BMI. However, an insulin resistance condition has not been reported in CD patients, neither at diagnosis nor after GFD.

A GFD alters certain cardiovascular risk factors in CD patients, but the overall effect on cardiovascular risk in individuals remains unclear [59]. Therefore, it is important to explore the relationship between CD and cardiovascular disease. 

In a systematic review with meta-analysis, increased risk of stroke (OR 1.11; 95% CI 1.02–1.20; *p* = 0.316) in CD patients was reported, whereas a statistically significant increased risk of myocardial infarction and cardiovascular death was not found [60].

Moreover, a study done on a UK population did not reveal an increased risk of cardiovascular death in celiac patients compared to the general population (standardized mortality ratio: 1.23; 95% CI 0.98–1.51). However, the standardized mortality ratio was greater during the post-diagnosis period (after 2 years) than during the peri-diagnosis period (within 2 years) in CD patients [61].

In conclusion, taking into account the worse lipid profiles, the increases in new diagnoses of NAFLD, and the weight gain seen in CD patients (in particular in normal/overweight celiac patients), celiac patients need to be informed about these potential risks and counseled on personalized nutrition after starting a GFD. 

Despite a GFD being the only effective treatment available for celiac disease–and despite it playing a key role in mucosal healing and the remission of symptoms–liver functioning, body weight, and nutritional profiles should be monitored for the duration of a GFD.

## Figures and Tables

**Table 1:** Body mass index (BMI) values of celiac patients before and after starting a gluten-free diet (GFD)**.**

Study (Year)	Study Design	Follow-up Length	BMI Categories	Number of Patients before the Start of a GFD N(%)	Number of Patients after the Start of a GFD N(%)	BMI before the GFD	BMI after the GFD	*P*-Value
Barone et al. (2016) [29]	Prospective cohort	2.02 years (1.68–2.9 years) **	Obese ≥ 30	3 (7.7)	3 (7.7)	38 (36.2—39.4) **	35 (33.1—36.7) **	0.31
			Overweight 25–29.9	9 (23)	8 (20.5)	25.2 (25.2—26) **	25.2 (24.5–26) **	0.47
			Normal 18.5–24.9	23 (59)	26 (66.6)	20.7 (20.4—21.7) **	22.5 (21.1—23.1) **	<0.002
			Underweight < 18.5	4(10,3)	2(5.1)	18 (17,6—18) **	18 (17.7—18.2) **	0,41
			Total	39 (100)	39 (100)	21.5 (20.4–25.1) **	22.6 (21.1–25.2) **	0.07
Cheng et al. (2010) [11]	Retrospective cohort	2.8 years (2.7) *	Obese ≥ 30	25 (6.8)	20 (6.47)	33.8 (3.89) *	33.3 (3.9) *	<0.0001
			Overweight 25–29.9	56 (15.2)	52 (16.82)	27.1 (1.3) *	26.8 (2.4) *	
			Normal 18.5–24.9	224 (60.7)	194 (62.78)	21.4 (1.7) *	21.7 (2.3) *	
			Underweight < 18.5	64 (17.3)	43 (13.92)	17.3 (0.9) *	19 (2.7) *	
			total	369 (100)	309 (100)	22.4 (4.5) *	-	
Kabbani et al. (2012) [30]	Retrospective cohort	3.29 years (0.08–28.75) *	Total	679	679	24.0 (5.1) *	24.6 (5.1) *	<0.001
Stein et al. (2016) [31]	Case–control	At least 4 weeks	Total	107	107	23.8 (21.5–27.5) **	25.1 (21.6–28.8) **	≤0.001
Tortora et al. (2015) [32]	Prospective cohort	1 year	Total	98	98	22.9 (±4) *	24.1 (±4) *	<0.01
Tortora et al. (2018) [33]	Case–control	4.7 years (1.2) *	Total	370	370	23.2 (±3.6) *	24.8 (±3.5) *	<0.001

Note: * Values are expressed as the mean (SD); ** values are expressed as the median (IQR).

**Table 2:** Lipid profile in celiac patients before and after GFD.

Study (Year)	Study Design	Follow-up	Triglycerides mg/dl		Total Cholesterol mg/dl		LDL mg/dl		HDL mg/dl	
			Before GFD	After GFD	Before GFD	After GFD	Before GFD	After GFD	Before GFD	After GFD
Tortora (2015) * [32]	Prospective cohort	1 year	89.2(±40) (*n* = 98) p: 0.6	92.8(±50) (*n* = 98)	-	-	-	-	51.8(±14) (*n* = 98) *p*: 0.1	53.8(±14) (*n* = 98)
Tortora (2018) * [33]	Case-control	4.7(SD ± 1.2) Years	90.1(±37.8) (*n* = 370)	121(±50.3) (*n* = 370)	163.7(±35.2) (*n* = 370)	179.4(±29.3) (*n* = 370)	117.2(±39.1) (*n* = 370)	119.8(±38.1) (*n* = 370)	54.4(±13) (*n* = 370)	54.2(±12.8) (*n* = 370)
Ciccone (2019) ** [35]	Retrospective cohort	7 years (Range 1–36)	127.0(60-321) (*n* = 26) *p*: 0.0006	163.0(151-332) (*n* = 26)	191(90.0-322) (*n* = 88) *p*:0.0001	228(201-33) (*n* = 88)	-	-	F = 48(27.0—70.0) (*n* = 70) *p*:0.0001 M = 52(34.0-67.0) (*n* = 16) *p*:0.0001	F = 35(19—49) (*n* =7 0) M = 33(26—59) (*n* = 16)

Note: CD, Celiac Disease; GFD: Gluten-free diet; SD, Standard Deviation * Mean (±SD); ** Median (Range).

**Table 3:** The prevalence of type 2 diabetes (age ≥ 30 years) in celiac disease and control groups.

Study (Year)	Study Design	CD Group *N* (%)	Control Group *N* (%)	*P*-Value
Kabbani et al. (2013) [22]	Cross-sectional	26/840 (3.1)	81/840 (9.60)	<0.0001
Reilly et al. (2015) [36]	Prospective cohort	818/26,816 (3.1)	3919/130,051 (3)	-

Note: CD, celiac disease.

**Table 4:** Diagnosis of nonalcoholic fatty liver disease (NAFLD) and hepatic steatosis (HS) in celiac patients on a GFD.

**Study (Year)**	**Study Design**	**Follow-up**	**NAFLD/Celiac Disease**	**NAFLD/Healthy Control**	**OR, HR, *P*-Value**
Reilly et al. (2015) * [36]	Prospective cohort	11.4 ± 6.4 years	53/26861 (21/100.00) †	85/130051 (6/100.000) †	HR = 2.8 (2.0–3.8; *p* < 0.001)
Tovoli et al. (2018) [37]	Case–control	-	70/202 (34.7%)	44/202 (21.8%)	OR = 2.90 (CI: 1.64–5.15; *p* < 0.001)
**Study (Year)**	**Study Design**	Follow-up Years * (Range)	**HS/Celiac Disease before GFD**	**HS/Celiac Disease after GFD**	***P*-Value**
Ciccone et al. (2019) ** [35]	Retrospective cohort	7(1–36) years	3/185 (1.7%)	20/185 (11.1%)	*p* < 0.0001
Imperatore et al. (2018) * [38]	Prospective cohort	1 years	78/301 (25.9%)	112/301(37.2%)	*p* < 0.01

Note: CD: celiac disease; NAFLD: nonalcoholic fatty liver disease; HS: hepatic steatosis; HR: hazard ratio; OR: odds ratio; HS: hepatic steatosis; * (mean ± SD); ** median; † person years.

## Appendix A

**Table A1:** Baseline characteristics of the included studies.

Study	Study Design	Patients	Age	Control Group	Clinical and Metabolic Findings Assessed
Barone et al. (2016), Italy [29]	Prospective cohort	patients with CD (*N*: 39)	≥18	39	BMI
Cheng et al. (2010), USA [11]	Retrospective cohort	patients with CD (*N*: 369)	≥18	not any	BMI
Kabbani et al. (2012), USA [30]	Retrospective cohort	patients with CD (*N*:679)	≥18	not any	BMI
Stein et al. (2016), USA [31]	Case–control	patients with CD (*N*:174)	≥17	696	BMI
Tortora et al. (2015), Italy [32]	Prospective cohort	patients with CD (*N*:98)	≥18	not any	BMI, dyslipidemia, T2DM
Tortora et al. (2018), Italy [33]	Case–control	patients with CD (*N*:136)	≥18	CD without HS (*N*: 234)	BMI, dyslipidemia, T2DM
Ukkola et al. (2012), Finland [34]	Prospective cohort	patients with CD (*N*:698)	≥16	3186	BMI
Dickey et al. (2006), UK [4]	Retrospective cohort	patients with CD (*N*:371)	≥18	not any	BMI
Ciccone et al. (2019), Italy [35]	Retrospective cohort	patients with CD (*N*:185)	≥18	not any	BMI, dyslipidemia, HS
Kabbani et al. (2013), USA [22]	Cross-sectional	patients with CD (*N*:840)	≥18	840	Dyslipidemia, T2DM
Reilly et al. (2015), Sweden [36]	Prospective cohort	patients with CD (*N*:26,816)	≥18	130,051	T2DM, NAFLD
Tovoli et al. (2018), Italy [37]	Case–control	patients with CD (*N*:2020)	≥18	202	NAFLD
Imperatore et al. (2018), Italy [38]	Prospective cohort	patients with CD (*N*:301)	≥18	GERD (*N*:50)	HS
Kamal et al. (2018), Egypt [19]	Prospective cohort	patients with HS (*N*:2594)	≥18	NAFLD without CD (*N*:100/2594)	NAFLD

Note: CD, celiac disease; GFD, gluten-free diet; BMI, body mass index; HS, hepatic steatosis; T2DM, type 2 diabetes mellitus; GERD, gastroesophageal reflux disease; NAFLD, Nonalcoholic fatty liver disease.
